# 

**Published:** 1894-10

**Authors:** 


### CONTENTS:

ORIGINAL ARTICLES: PAGE

The Anatomy of the Large American Fluke (*Fasciola Magna*). By Charles Wardell Stiles, Ph.D. .... 299
The Influence of Climate and other Environments on the Distribution and Character of Disease. By W. L. Williams, V. S. .... 313
How can we Control the Prevalence of Tuberculosis among Cattle. By M. R. Trumbower, D. V. S. .... 335
Diseases of the Liver as a Direct Cause of Intestinal Disturbances. By Dr. D. F. Fox. 340
An Act to Establish a Live-stock Commission, etc. (Pennsylvania) .... 344
The Advantages of the Point System of Judging, and how it Should be Initiated .... 348

EDITORIAL:

McKillip Veterinary College .... 35

SOCIETY PROCEEDINGS:

Maine Veterinary Medical Association .... 354
California State Veterinary Medical Association .... 357
The Maryland State Veterinary Medical Society .... 359

Items .... 364

